# Statistics of work and orthogonality catastrophe in discrete level systems: an application to fullerene molecules and ultra-cold trapped Fermi gases

**DOI:** 10.3762/bjnano.6.78

**Published:** 2015-03-18

**Authors:** Antonello Sindona, Michele Pisarra, Mario Gravina, Cristian Vacacela Gomez, Pierfrancesco Riccardi, Giovanni Falcone, Francesco Plastina

**Affiliations:** 1 Dipartimento di Fisica, Università della Calabria, Cubo 30C, 87036 Rende (CS), Italy; 2 INFN, sezione LNF, Gruppo collegato di Cosenza, Cubo 31C, 87036 Rende (CS), Italy

**Keywords:** nanostructured systems, non-equilibrium thermodynamics, orthogonality catastrophe, sudden quench, ultra-cold Fermi gases, work distribution

## Abstract

The sudden introduction of a local impurity in a Fermi sea leads to an anomalous disturbance of its quantum state that represents a local quench, leaving the system out of equilibrium and giving rise to the Anderson orthogonality catastrophe. The statistics of the work done describe the energy fluctuations produced by the quench, providing an accurate and detailed insight into the fundamental physics of the process. We present here a numerical approach to the non-equilibrium work distribution, supported by applications to phenomena occurring at very diverse energy ranges. One of them is the valence electron shake-up induced by photo-ionization of a core state in a fullerene molecule. The other is the response of an ultra-cold gas of trapped fermions to an embedded two-level atom excited by a fast pulse. Working at low thermal energies, we detect the primary role played by many-particle states of the perturbed system with one or two excited fermions. We validate our approach through the comparison with some photoemission data on fullerene films and previous analytical calculations on harmonically trapped Fermi gases.

## Introduction

Closed many-particle systems and their out-of-equilibrium dynamics after a quench have been attracting considerable interest over the past years, with particular attention to the brutal disturbance of the equilibrium properties of a Fermi gas, induced by the sudden introduction of localized scattering potential in the system [1–4]. Notwithstanding the weakness of the perturbation, its effect can be so pronounced that the final state of the gas loses essentially any overlap with the initial unperturbed one, as the number of particles approaches the thermodynamic limit. This *orthogonality catastrophe* predicted by Anderson [5] was first witnessed by the anomalous response of conduction electrons to core level ionization through X-ray absorption, and the subsequent emission of a core electron from simple metals [6–7]. The corresponding kinetic energy spectrum was observed to have an asymmetric peak at the binding energy of the core level with a power-law singularity, which has then become known as the *Fermi edge singularity* [7]. Similar patterns were afterwards identified in a large number of core-ionized systems [8–9], including organic molecules [10] and carbon-based nanomaterials [11–19], where an additional signature of the Anderson orthogonality catastrophe are the secondary peaks, or shake-up satellites, in the core level spectra. Despite the diversity of contexts in which Fermi edge resonance and Anderson orthogonality catastrophe occur [20–30], the same generic physics has been recently observed in the controllable domain of ultra-cold trapped gases, as a response to the embedding of a single probe qubit, i.e., a two-level impurity [31–32]. Furthermore the intrinsic out-of-equilibrium dynamics induced by the impurity has been thoroughly analyzed by treating the quench as a thermodynamic transformation [33], and using the full statistics of the work done on the gas [34–35]. Interestingly enough, X-ray absorption and emission spectra from noninteracting quantum dots have been interpreted in terms of the quantum work distribution, and linked to the corresponding fluctuation relations in statistical mechanics [36]. To explore more of such a connection, we present here a comparison of the statistics of the work done in the C_60_ molecule, following the core ionization of a carbon atom, and a harmonically trapped Fermi gas, following the sudden switch on of a localized perturbation, assumed to have an s-wave-like character. In particular, we propose a numerical approach suitable for low-temperature regimes to compute the work distribution (Section 1), based on the knowledge of the initial ground state and the low-lying final perturbed states of the systems (Section 2). To treat the fullerene molecule, we use density-functional theory (DFT) and simulate the sudden creation of a core state, by replacing a 1s electron pair with the effective pseudo-potentials of neutral and ionized atomic carbon (Section 2.1). In the harmonically trapped Fermi gas, on the other hand, we assume a contact scattering potential with a spatially structure-less form, localized at the center of the harmonic trap, and express the potential strength in terms of a dimensionless parameter, which turns out to be the critical parameter governing the sudden quench process (Section 2.2). We then determine the one-fermion structures of the systems in the absence or presence of the perturbation, and compute the many-body overlap between the initial unperturbed ground state and the final perturbed states, with not more than two excited fermions (Section 3). The work distribution obtained with such contributions accounts for more than 95% of the shake-up process, which let us select the suitable parameters in the Fermi gas with a shake-up content similar to that of fullerene. We test the accuracy of the methods through the comparison with available X-ray photoemission experiments [12–13], in the fullerene case, and previous analytical calculations [32,35], in the Fermi gas case. Finally, we draw some conclusions on the results obtained in the two applications (Section Conclusion).

## Results and Discussion

### 1 Work distribution and energy spectrum in a sudden quench

We begin by reviewing some concepts regarding non-equilibrium thermodynamics in a suddenly quenched Fermi gas. Consider a many-fermion system in a well-defined Gibbs state


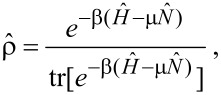


at inverse temperature β and chemical potential μ. The equilibrium is set by the initial Hamiltonian 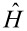 and the number operator 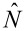, which are diagonal in the same basis of eigenstates 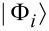 having the eigenvalues *E_i_* and *N_i_*, respectively. After removing the contact with the thermal reservoir, suppose some *work* is performed by taking the system out of equilibrium through the abrupt introduction of an external perturbation 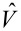. Now the perturbed system is characterized by the final Hamiltonian 
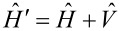
, specified by the eigenstates 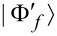 and the eigenenergies 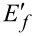. In this picture, the work done is not a quantum mechanical observable, but rather a stochastic variable distributed according to a probability distribution *P*_β_(*W*) [34]. The definition of such a distribution requires two projective measurements: the first projects onto the eigenbasis of the initial Hamiltonian, with the system in thermal equilibrium. The system then suddenly evolves, before the second measurement projects onto the eigenbasis of the final Hamiltonian. Accordingly, the probability to do the work 
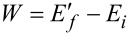
 is given by the probability 
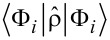
 of obtaining *E_i_* for the first measurement outcome, followed by the conditioned probability 
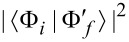
 of obtaining 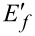 for the second. The work distribution is therefore obtained as [33]

[1]
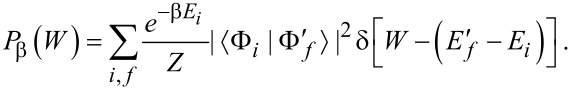


At the absolute zero, only the unperturbed ground state remains in the initial state summation

[2]
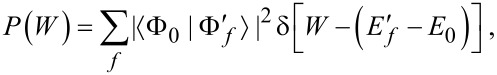


and the work distribution tends to the initial state average 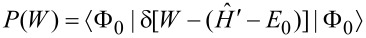, which coincides with the emission spectrum of the system in response to the sudden perturbation [37–38]. Interestingly enough, the cross-section for the ionization of a core level ε*_c_* due to absorption of an X-ray photon of energy 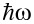 in matter results from two factors [39]: core-electron photo-ejection and valence-electron dynamic screening. The former is expressed by the photo-current probability, involving the initial core state and the final photo electron state. The latter is manifested by the work distribution in Equation 2, which can be interpreted as the probability density that the work 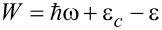 is used to excite valence electrons at the expense of the kinetic energy of the photoelectron, ε [17–19]. Indeed, *P*(*W*) accounts for the *N* − 1 electrons that do not directly participate in the ionization process. This *spectator electron* approach is particularly suitable for mono-energetic X-rays that cause deep core-level photoelectrons to be ejected from the sample (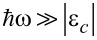).

### 2 Initial and final Hamiltonians

We have seen that the key ingredients of the work distribution (Equation 2) are the many-body states of the unperturbed and perturbed Fermi systems. In the following we will take the different physical situations set forth above. Specifically, we will first investigate the valence electronic structure of a fullerene molecule in the absence or presence of a 1s core hole, whose fast creation induces an abrupt attractive perturbation of the electrons of the system. Then, we will shift the focus to a harmonically trapped gas of fermionic atoms with an embedded impurity, whose fast excitation can be modeled by a suddenly introduced repulsive δ-potential.

#### 2.1 The fullerene molecule

**Figure 1:**
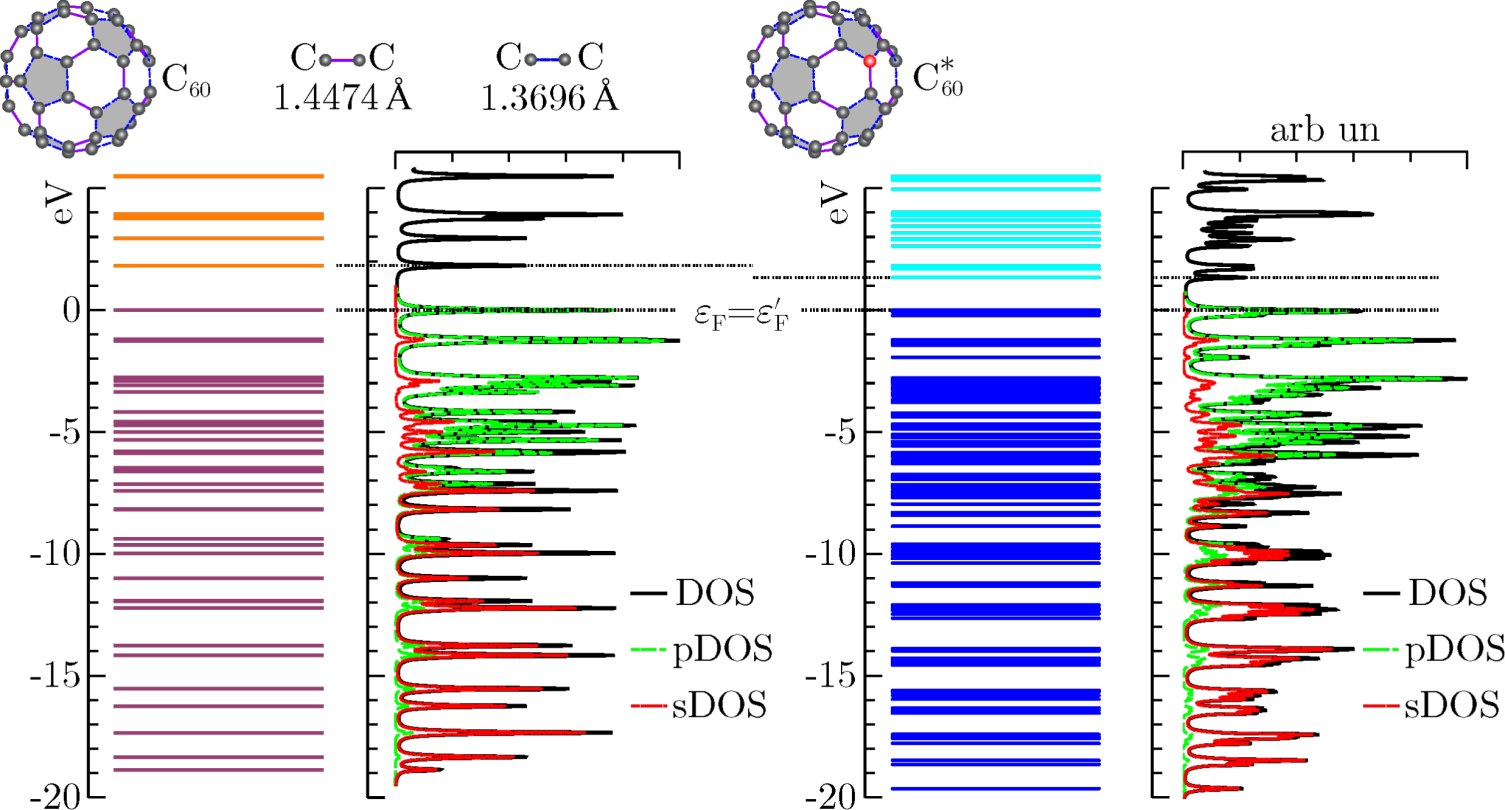
Valence levels and (broadened) DOS for the neutral (C_60_) and ionized (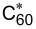) fullerene molecules, as computed with the DFT approach outlined in the main text. In C_60_ the projected density of s and p states contribute for 44.2% and 55.3% of the occupied DOS, respectively. In 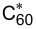 the s-DOS and p-DOS contributions change to 51.1% and 48.8%, respectively. The residual part (not shown) is left to the projected density of polarized d-states.

Consider a cluster of 60 carbon atoms arranged in a fullerene molecule of radius 3.1573 Å, whose equilibrium geometry and characteristic bond lengths (of 1.4474 and 1.3696 Å, respectively) are reported Figure 1. We can do some *work* on the cluster by core-ionizing one of its atoms to form a molecular cation. The valence electrons are then thrown out of equilibrium, tending to dynamically relax and compensate for the presence of a positive charge. To depict the rearrangement of the valence electronic structure, we use a DFT approach in which we replace the core electrons of a specific atom in the molecule with an effective core potential (ECP) of the Stevens–Basch–Krauss (SBK) type [40], whose parameters are adjusted to describe neutral and core-ionized atomic carbon [10]. The valence electrons in this reference atom are described by a d-polarized double split-valence pseudo-basis, being specifically designed for the considered ECP and optimized for the neutral (C_60_) and ionized (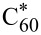) clusters [18–19]. As for the core and valence electrons of all other atoms in the compound, we select the d-polarized triple split-valence basis set denoted 6-311G* [41]. We then perform a spin-restricted DFT calculation [42–44], working under the generalized gradient approximation (GGA) for electron exchange and correlation, parameterized by the Perdew–Burke–Ernzerhof (PBE) functional [45–46]. All electron spin pairs from the clusters are explicitly taken into account except for the one removed from the reference atom. Convergence for C_60_ and 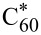 leads to optimized ground state wave functions made of single Slater determinants of 179 pairs of occupied molecular orbitals (MOs), which are linear combinations of 1135 contracted Gaussians from both the ECP basis, localized at the reference atom, and the 6-311G* basis centered on all other atoms. To simplify the notation we denote such composite basis sets as 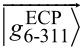 for C_60_, and 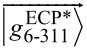 for 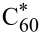. The corresponding coefficients (eigenvectors) are computed from the secular equations following DFT energy minimization.

The electron spin pairs occupy 59 core levels, that is, one per carbon atom excluding the reference atom, and 120 valence levels. The core MOs are mainly given by linear combinations of the s-contracted Gaussians of the ECP+6-311G* basis set, where the valence coefficients of the ECP set tend to compensate for the absence of the core electrons in the reference atom. The core eigenvalues are nearly degenerate with a percentage standard deviation below 0.15%. The average core energy ε*_c_* for C_60_ overestimates the experimental C 1s energy by a percentage error of about 7%. Possible causes for this discrepancy are discussed in [18]. The valence states are of the form 
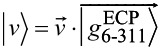
 and 
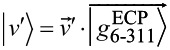
, where 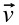 and 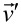 are the valence-eigenvectors of coefficients for C_60_ and 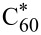, respectively. As shown in Figure 1, the valence electronic structure of the neutral and ionized clusters is made of discrete energy levels separated by an average energy difference of about 0.2 eV. The predicted band gap value of 1.82 eV for C_60_ is consistent with experiments [47] and previous calculations [18]. Core ionization leads to a decrease of the band gap in 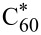 of about 0.5 eV (Table 1).

**Table 1:** Squared overlap integrals between the lowest and highest occupied valence states in the neutral C_60_ molecule (*v* = 1, ε_1_ = −18.8575 eV and *v* = *v*_F_ = 120, ε_120_ = ε_F_ = 0, respectively) and some valence states in the ionized C_60_ molecule (where 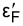 = −3.132 eV). The reported values confirm the remarkable non adiabatic effects induced by core hole creation (see also below in Figure 2).

*v*′	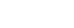	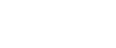	*v*′	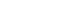	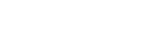

1	−19.6357	0.428741	115	−1.2163	0.000219
2	−18.6425	0.554975	116	−0.2204	0.791579
3	−18.4765	0.009811	117	−0.0762	<10^−6^
4	−18.4765	<10^−6^	118	−0.0653	0.004186
5	−17.7799	0.003615	119	−0.0163	<10^−6^
6	−17.5758	0.000141	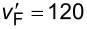	0	<10^−6^
7	−17.5214	<10^−6^	121	1.3361	0.042688
8	−17.4262	0.000096	122	1.6871	<10^−6^
9	−17.4126	<10^−6^	123	1.8096	0.000134
10	−16.5364	0.000784	124	2.6232	0.012468
11	−16.4248	0.000091	125	2.8799	<10^−6^

**Figure 2:**
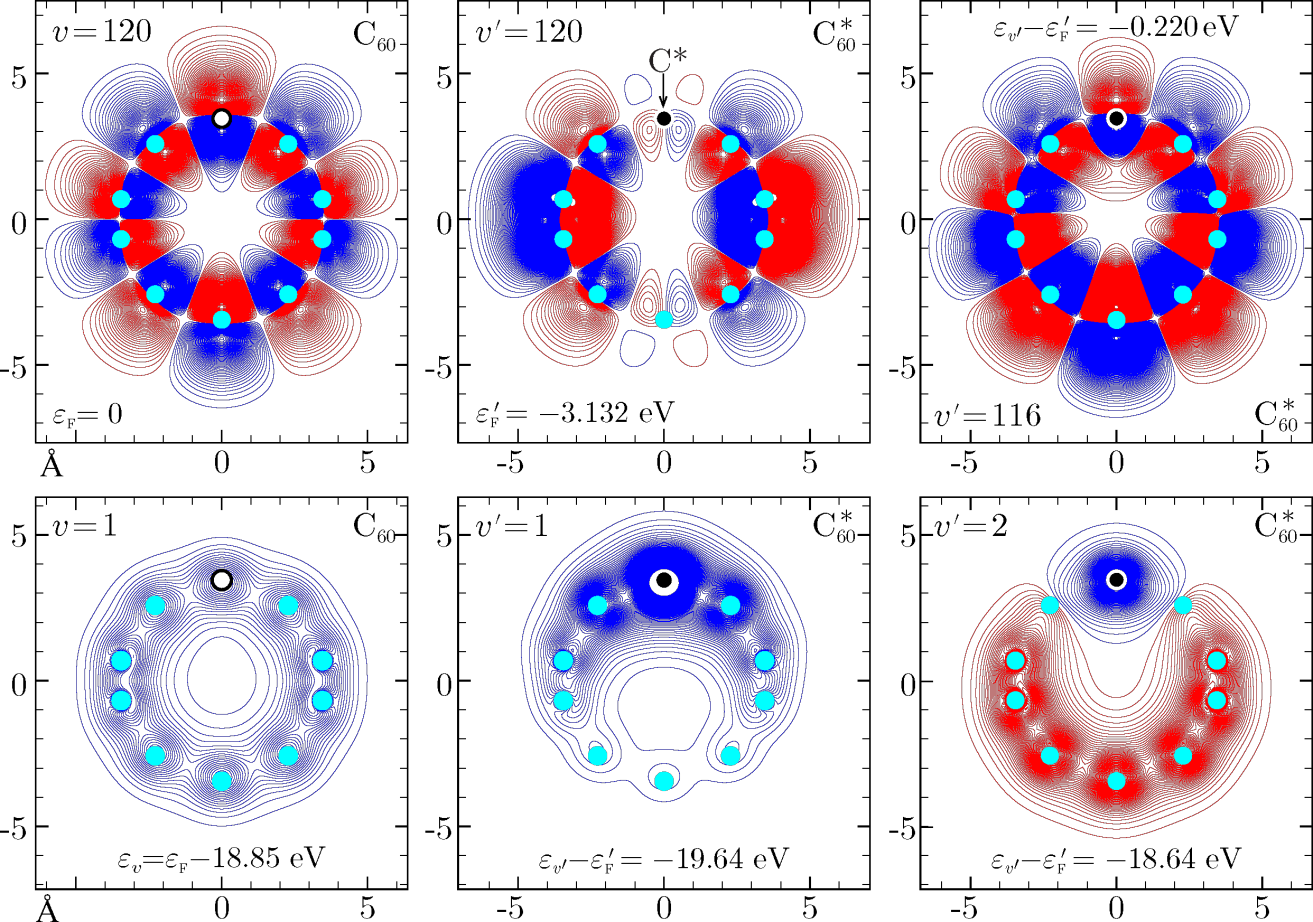
Lowest and highest occupied valence states for the neutral fullerene molecule (C_60_) and corresponding valence states in the ionized fullerene molecule (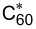). The sudden switching mechanism leads most of the content of the unperturbed Fermi state to be found in a state lying about 0.22 eV below the perturbed Fermi level. On the other hand, the lowest- energy unperturbed valence state gets mainly mixed with the first two occupied perturbed valence states (see also Table 1).

In order to determine the symmetry of the valence MOs, we compute a density of state (DOS) distribution from the superposition of Lorentzian lines of equal height, centered at the occupied/empty MO energy values. We then use the valence coefficients to construct a weighted sum yielding the *projected* distributions arising from the s, p, and d components of the ECP+6-311G* basis set. The normalized profiles of the total DOS, the s-DOS, and the p-DOS for both the neutral and ionized molecules are also displayed in Figure 1, where a broadening width of about 0.5 eV is applied. We see that the lowest occupied valence MOs, with energies in the range of ca. 10–20 eV below the Fermi level, have a dominant s character. At higher energies, up to about 2–3 eV below the Fermi energy, the p components become more and more significant, tending to compete with the s components and forming sp^2^ and sp^3^ bonds. On the other hand, the valence MOs close to the Fermi energy are mainly made of p orbitals pointing along the radial directions of the buckyball. Based on the analysis of the relative areas of the projected densities of states, we may infer that core ionization produces an enhancement of the s component with respect to the p component of about 5%, while polarization effects due to the d orbitals play a marginal role. This is not surprising given the s character of the core hole. The key feature of the many-electron response to core ionization is given by the squared overlaps between the valence MOs of C_60_ and 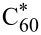. The latter are straightforwardly computed from the 1135 × 1135 overlap matrix 
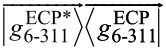
, by left (right) multiplication with the valence eigenvectors 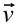 and 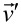, respectively. To have a more clear idea of the change of the valence electrons wavefunctions in the surrounding of the core-hole site, we focus on the ends of the occupied valence spectra. In particular, we consider the highest and lowest occupied MOs of the neutral molecule and some MOs of the ionized molecule to have similar binding energies relative to the perturbed Fermi level. The squared overlap between these states are listed in Table 1, while some of their orbital shapes are shown in Figure 2.

We see that core ionization has more direct influence on the bottom of the valence band, inducing the lowest occupied state 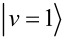 of C_60_ to get mostly mixed with the first two occupied states of 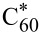, namely 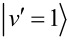 and 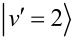. Significant modifications, however, affect also the unperturbed Fermi state 
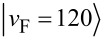
, which looses essentially any correlation with the perturbed Fermi state 
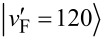
, and is mainly mapped to the 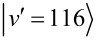-state keeping some non negligible leakage to some other perturbed states with similar energies. This is a clear signature of the highly non-adiabatic behavior inherent to the process. As a global measure of the disturbance brought by the core hole, we take the valence electron ground states 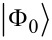 and 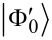, for the neutral and ionized molecule, respectively, and compute their squared overlap


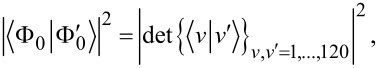


which denotes the ground-state survival probability. Complementarily, the shake-up probability is given by 
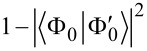
 and takes a value of 19.10%.

#### 2.2 The harmonically trapped Fermi gas

We now take a spin 1/2 gas of weakly interacting atoms in a parabolic potential of a typical length *x*_0_ and trapping frequency ω. Neglecting the inter-particle forces, the one-fermion Hamiltonian is that of a harmonic oscillator

[3]
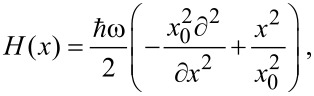


with eigenvalues 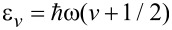, and eigenstates 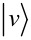, which have the coordinate representation

[4]
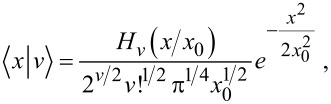


expressed in terms of the Hermite polynomials *H_v_*(*x*/*x*_0_) of order *v* = 0,1,…. We now add a two-level impurity trapped in an auxiliary potential and brought in contact with the gas. The impurity is initially in its ground state with a negligible scattering interaction with the fermions in their equilibrium configuration set by *H*(*x*). We suppose doing some *work* on the system by quickly exciting the impurity. Then, the gas feels a sudden perturbation *V*(*x*,*t*) = *V*(*x*)θ(*t*), assumed to have an s-wave like character. Further details on how this set-up can efficiently describe an ultra-cold Fermi gas probed by a two-level impurity, with the parabolic potential mimicking the magneto-optical trapping potential, may be found for example in [25,28,31–32,48]. Let us further assume that the perturbation is spatially structure-less and localized at the center of the trap, e.g., *V*(*x*) = π*V*_0_*x*_0_δ(*x*). The perturbation strength *V*_0_ can be parameterized as 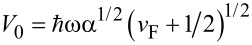, where *v*_F_ is the Fermi number (corresponding to 2(*v*_F_ + 1) fermions, and α a dimensionless parameter, which turns out to be the critical parameter of the theory [32,35,49].

The simple structure of *V*(*x*) allows one to handle the diagonalization of the total Hamiltonian *H*′(*x*) = *H*(*x*) + *V* (*x*), which describes the gas after switching on the potential. In particular, the perturbed eigenfunctions in presence of the excited impurity can be written in terms of the parabolic cylinder functions

**Figure 3:**
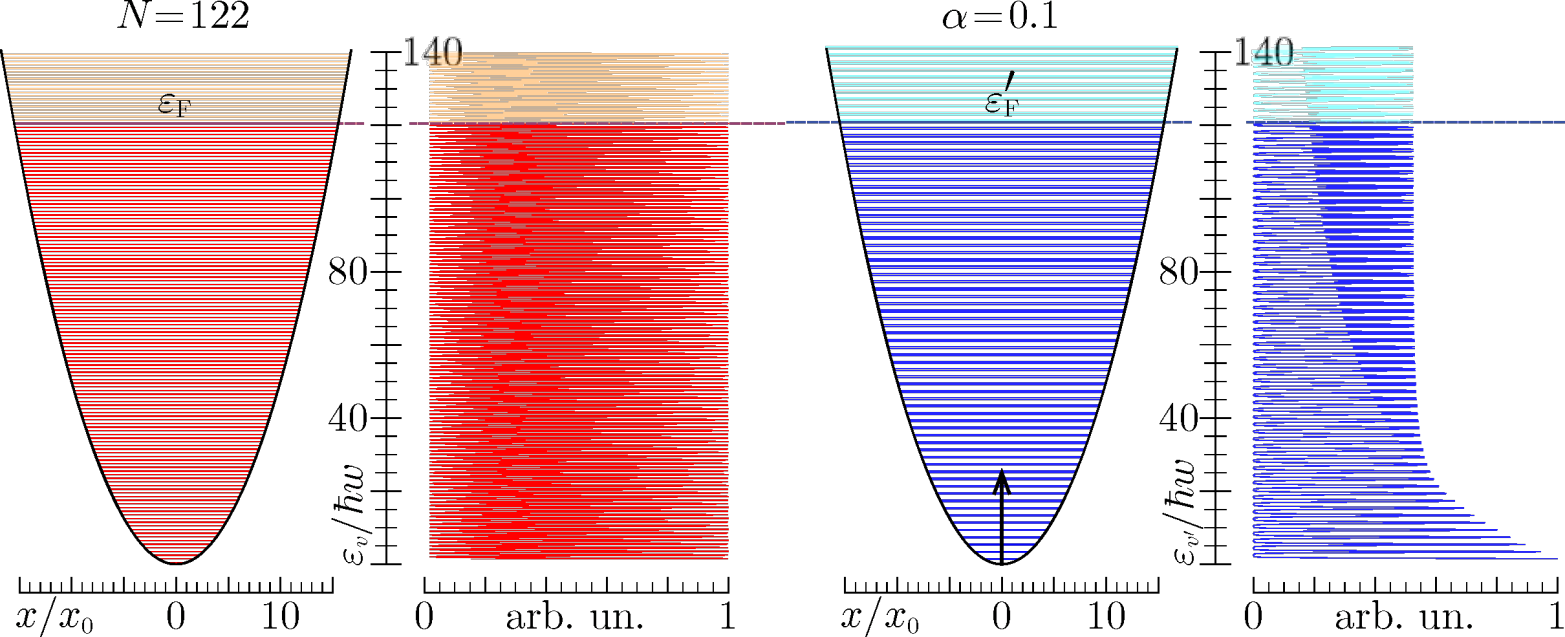
Energy levels and (broadened) density of states for a trapped gas of spin 1/2 fermions having a number of occupied states (*N* = 122) similar to that of the valence band of C_60_. The critical parameter is set to α = 0.1, which corresponds to an impurity potential height of 
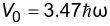
.

**Figure 4:**
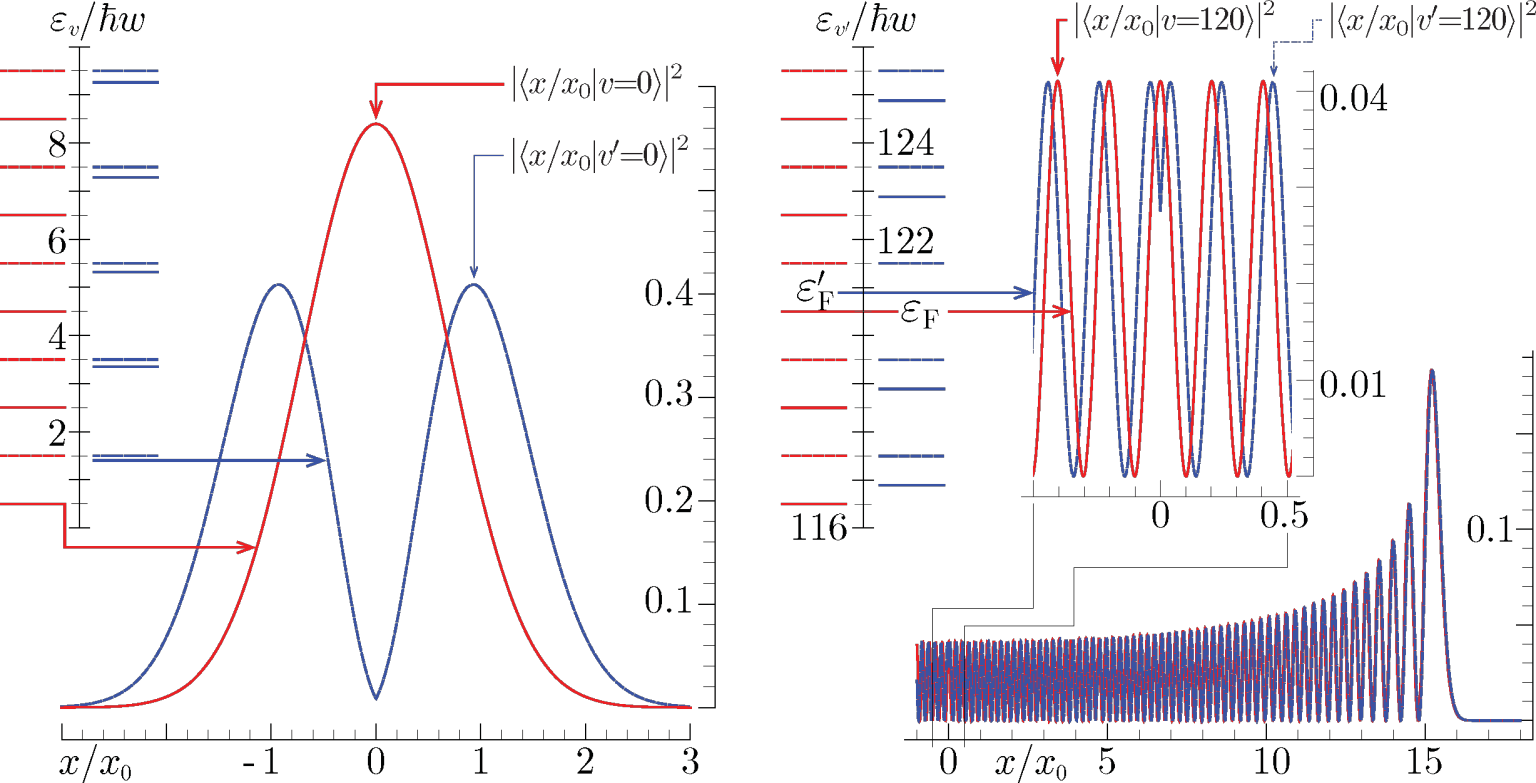
Lowest and highest occupied one-particle wave functions and levels for a harmonically trapped gas of *N* = 122 fermions in absence and presence of the excited impurity, whose perturbation is characterized by the critical parameter α = 0.1 (see also Figure 3 and Table 2). The unperturbed and perturbed Fermi levels, relative to the lowest occupied one-particle state, are 
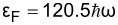
 and 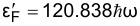, respectively.

[5]
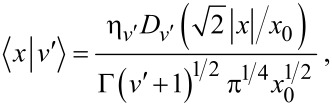


with normalization constants η*_v_*_′_ and associated level energies 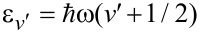. We see that 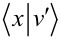 = 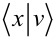 and ε*_v_*_′_ = ε*_v_* when the perturbed quantum number *v*′ takes non-negative integer values. Furthermore, due to the fact that 
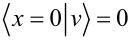
, for *v* = 1,3,…, the odd harmonic oscillator eigenfunctions and eigenenergies are left unaffected by the δ-potential, i.e., *v*′ = *v* = 1,3,…. As for the perturbed wave functions corresponding to *v* = 0,2,…, the stationary Schrödinger equation for *H*′(*x*) leads the implicit condition [35,50]:

[6]
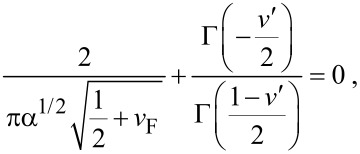


which ensures the physically correct behavior for 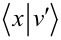. Now, since the Γ-function has poles for negative integer values, Equation 6 leads to *v*′→*v* for α→0, and *v*′→*v* + 1 for α→∞. Then, *v*′ takes a real values in the range [*v*,*v* + 1] for *v* = 0,2,…. More importantly, each fixed values of α and *v*_F_ yields a one-to-one mapping of 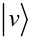, ε*_v_* onto 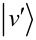, ε*_v_*_′_. This means that we can first obtain the *v*′ values by numerically solving Equation 6, compute the perturbed energies ε*_v_*_′_ and the normalization constants η*_v_*_′_, and then find the perturbed states 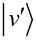. In Figure 3 and Figure 4 we show an example of gas with 122 fermions (*v*_F_ = 120) in absence and presence of an impurity potential characterized by the critical parameter α = 0.1. Similar to the fullerene case, we see that the sudden perturbation is more efficient on the lower part of the energy spectrum, which corresponds to a more pronounced shifting of the perturbed even levels towards the unperturbed odd ones. This is also attested by comparing the unperturbed and perturbed density of levels, obtained by superimposing Gaussian functions of width 0.15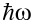, centered at the occupied/empty energy values. A more quantitative analysis comes from the squared overlaps 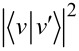, some of which are numerically computed and reported in Table 2. In contrast to the C_60_/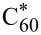 case, we notice that the states involved is the ε*_v_*,
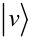
↔ε*_v_*_′_,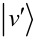 mapping are always strongly correlated by a squared overlap value larger than 
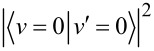
≈ 0.74.

**Table 2:** Squared overlap integrals in a spin 1/2 trapped gas of 122 particles with a sudden switching impurity potential characterized by the critical exponent value α = 0.1 (see also Figure 3). The lowest occupied (*v* = 0, ε_0_ = 0.5 eV) and highest occupied (*v* = 120, ε_120_ = ε_F_ = 120.5) states are mostly correlated to the corresponding perturbed states. Shake-up effects though involve the perturbed states which are closer in energy.

*v*′	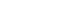	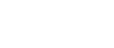	*v*′	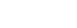	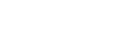

0	1.38233	0.742291	110	110.85	0.00147324
2	3.32392	0.108816	112	112.848	0.00234289
4	5.28188	0.046455	114	114.845	0.00429914
6	7.24826	0.0265519	116	116.843	0.010353
8	9.22	0.017436	118	118.84	0.0518994
10	11.1955	0.0124231	120	120.838	0.882784
12	13.1739	0.00934134	122	122.836	0.0234252
14	15.1546	0.00729833	124	124.834	0.00692288
16	17.137	0.00586813	126	126.831	0.00325807
18	19.121	0.00482476	128	128.829	0.00188445
20	21.1063	0.00403857	130	130.827	0.00122525

Nonetheless, a much more regular dynamic screening is experienced by the gas, involving single fermion states with squared overlaps 
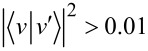
. Indeed we observe a non-negligible leakage of the unperturbed wave function onto the perturbed eigenfunctions, having an energy larger than 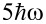 than the unperturbed energy value. With the two eigenbases 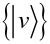 and 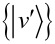, we can form Slater determinants and compute the many-body states of the gas. In particular, the unperturbed and perturbed ground states include the lowest occupied 2(*v*_F_ + 1) one-particle states, and the ground-state survival probability can be computed from


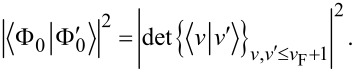


The shake-up probability 
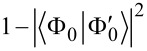
 increases with increasing height of the impurity potential barrier. In the example considered here (Figure 3 and Figure 4) this probability takes a percentage value of 19.26%, which is extraordinarily similar to that of fullerene. Suppose we keep the critical exponent constant, i.e., we fix α = 0.1, but reduce the number of fermions in the gas to *N* = 38 first, and then to *N* = 16. The corresponding shake-up probabilities will decrease to 15.36% and 12.17%, respectively. Suppose, as a complement, we keep the particle number constant, say, *N* = 122, and increase α to 0.2 first, and then to 0.3. The corresponding shake-up probabilities will increase to 29.3% and 35.7%, respectively.

### 3 Work distribution decomposition and many-fermion shake-up

To characterize the zero-temperature features of the work distribution (Equation 2), we decompose it according to the number of fermions excited to the final states by the external potential. In other words, we re-arrange the final state summation in Equation 2 to write it in the form


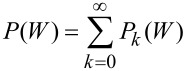


where *P_k_*(*W*) accounts for the work done in all processes which lead the system to occupy a final state with *k* fermions above the Fermi level. Now, we use the formalism of creation and annihilation operators, denoted 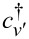 and *c_v_*_′_, respectively, acting on the perturbed ground state, to express the *P_k_*(*W*) distributions as shown in Equation 7 (see below).

[7]
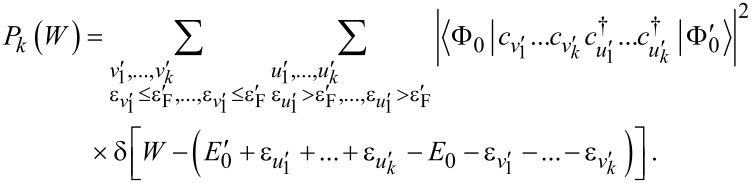


Here, the squared overlaps can be reduced to the calculation of matrix determinants involving the unperturbed and perturbed one-fermion eigenstates of the initial and final Hamiltonians, i.e., the unperturbed ground state set 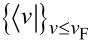 and the perturbed set obtained by taking 
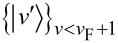
 and replacing the elements 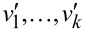
with 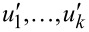. We also need to point out that in the work distribution of fullerene we do not include the core electrons, which do not take part in the photo-ejection. Indeed, the overlap between the initial and final many-body states of the “spectator” core levels is 1, within a numerical error of ca. 10^−6^. This fact is not surprising, notwithstanding the differences in the one-electron core states (Figure 2), because excitations from the core to the valence part of the one-electron initial and final spectra are not allowed.

The situations that we have considered so far encompass relatively weak external perturbations, for which the most prominent contribution is the no-shake line

[8]
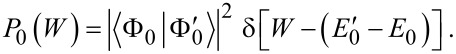


This term corresponds to a process in which the work 
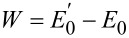
 is used to distort the initial ground state, and leave all the particles in the system relaxed into the final ground state. Indeed, as already pointed out in the previous section and emphasized by the results shown in Table 3, the no-shake intensity, i.e., the ground-state survival probability, takes percentage values of the order of about 80% either in the core-ionized fullerene molecule or in the Fermi gas with *N* = 122 particles, shaken up by a δ-potential of critical exponent α = 0.1. The *P_k_*_≥1_ distributions define the shake-up process, with *k* fermions jumping between the (unperturbed) ground state and the (perturbed) excited states in response to the perturbation [35].

**Table 3:** Ground-state survival probability and shake-up probabilities involving excited states with 1–3 particles above the Fermi level. The closure relation 
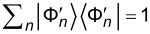
 , projected onto the unperturbed ground state, is verified with an error of less than 5% in all cases.

	C_60_	α = 0.1	α = 0.1	α = 0.1	α = 0.1
		*N* = 122	*N* = 84	*N* = 38	*N* = 16

no shake (%)	80.899	80.739	81.876	84.635	87.834
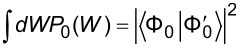					
one body (%)	17.031	14.975	13.736	11.614	8.091
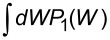					
two body (%)	0.249	0.211	0.147	0.067	0.012
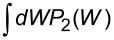					
three body (%)	≈10^−3^	≈10^−5^	≈10^−7^	≈10^−7^	≈10^−8^
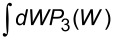					
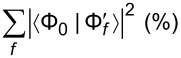	98.450	95.929	95.759	96.317	95.937
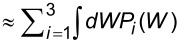					

**Figure 5:**
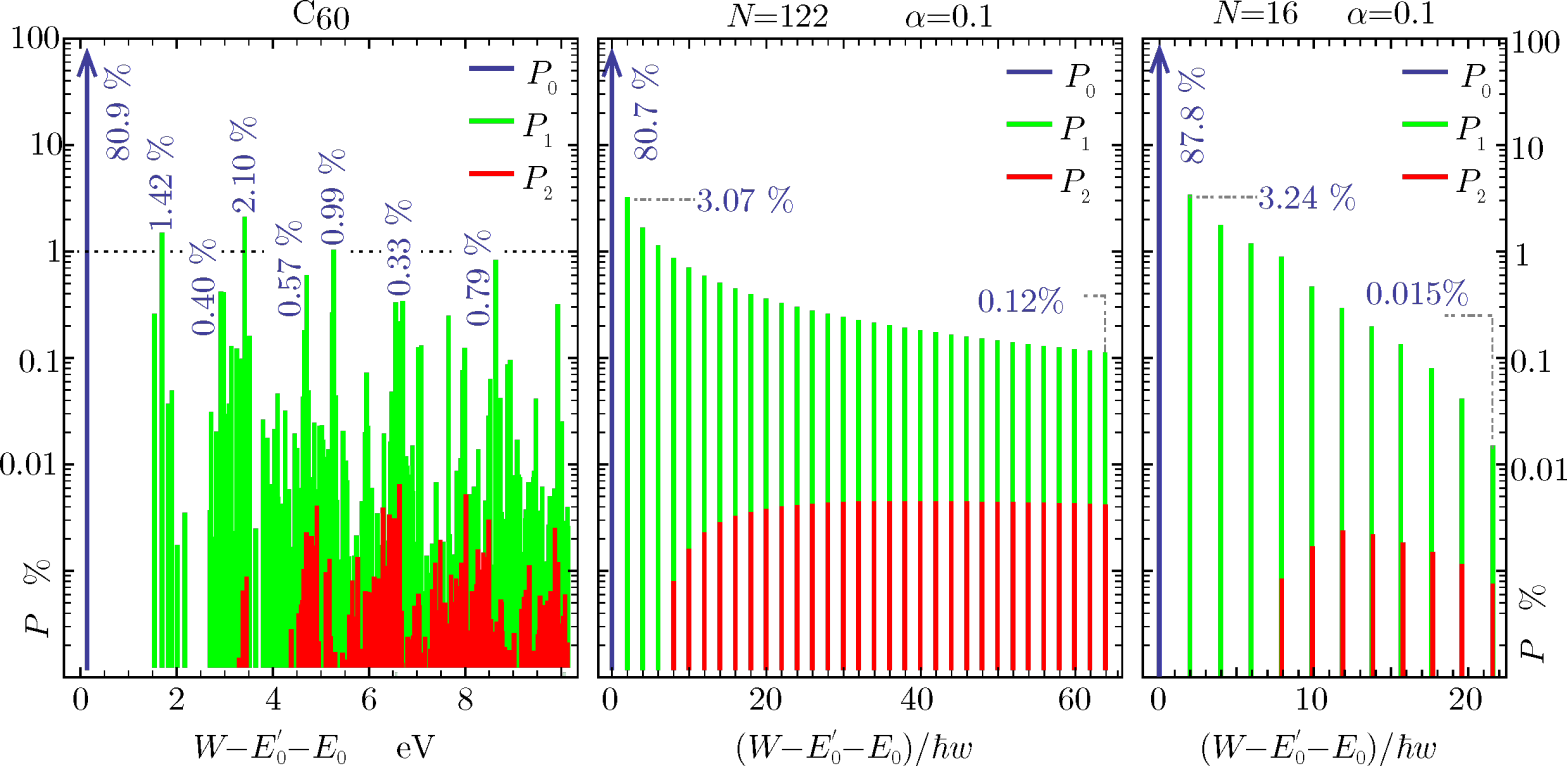
Zero-temperature work-distribution components (Equation 7) for a C_60_ molecule undergoing core ionization, and a Fermi gas of *N* = 16, 122 particles, shaken-up by a perturbation of critical index α = 0.1. Vertical values are give in percent, following Table 3.

Also from Table 3, we can see that the largest part of the fermion shake up lies in one-fermion excitations processes, e.g., in *P*_1_(*W*), while a residual contribution is from two-fermion excitations, included in *P*_2_(*W*), and three-fermion shake up may be generally neglected. The unitarity relation


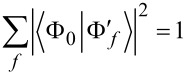


is verified within ca. 95–98% by restricting the *f*-summation to final states involving not more than two electrons excited at the considered energies. These features are supported by the plots in Figure 5, where we show how the partial components *P*_0_, *P*_1_ and *P*_2_ contribute to the zero-temperature work distribution (Equation 2). Besides the primary line, i.e., the no-shake intensity, we observe a sequence of secondary lines accounting, respectively, for one- and two-fermion transitions that are separated by 1–2 order of magnitudes. The three-fermion response lines (not shown) have maximum intensities smaller than 10^−5^% and 10^−7^%, in the two cases discussed here. Not visible enough, the shake lines of the harmonically trapped Fermi gas are almost uniformly spaced in steps of 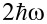. The non-perfect periodicity is due to the slight changes in the perturbed one-fermion energies (see also Table 2), which where not caught by the perturbation model of [32,35].

To finalize the analysis, we briefly discuss how to include temperature effects into Equation 2, approximated as


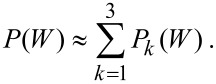


**Figure 6:**
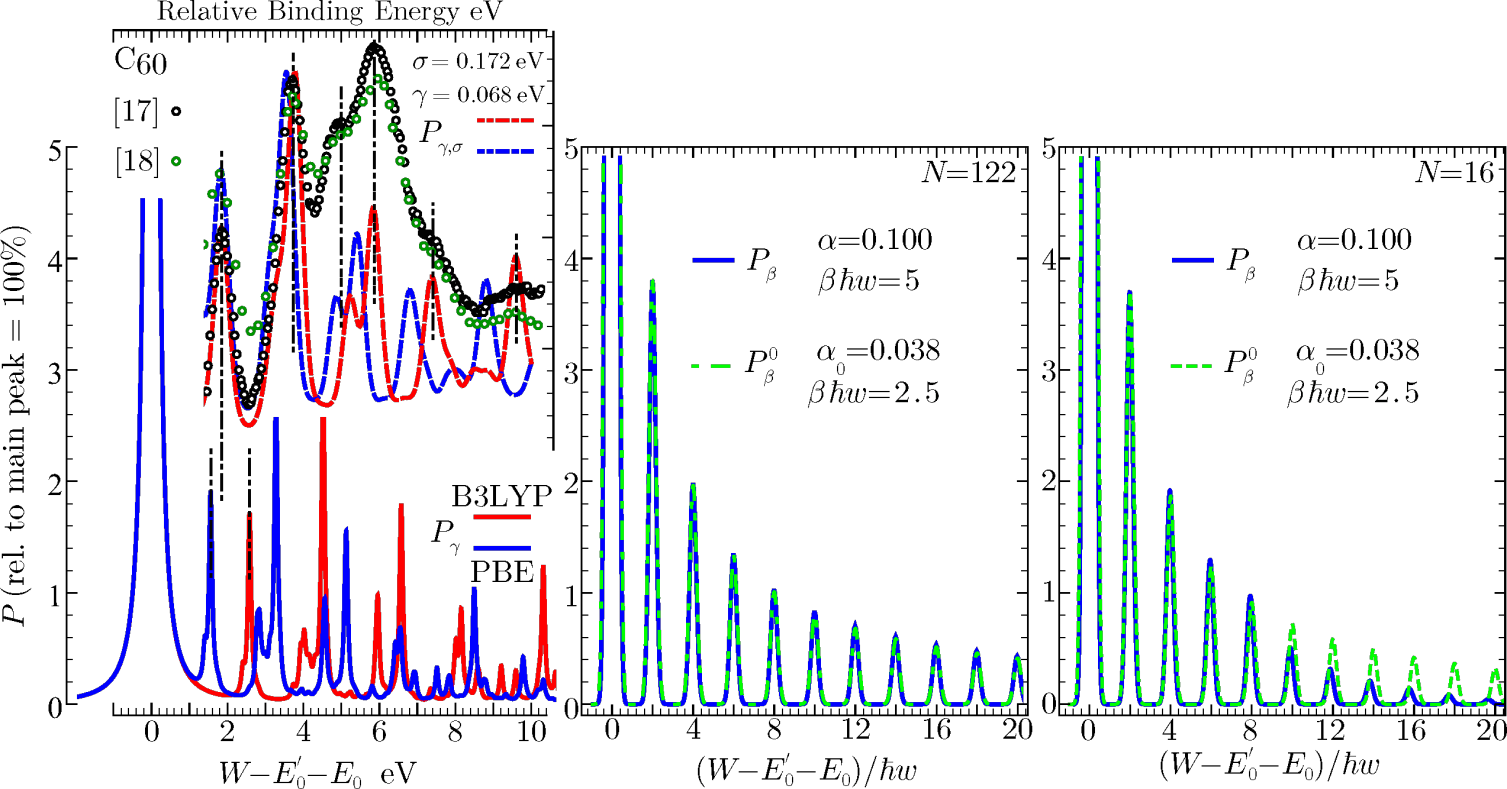
Low-temperature work distributions for: (left) a C_60_ molecule undergoing core-ionization; (center,right) a Fermi gas of *N* = 16, 122 particles shaken-up by a perturbation of critical index α = 0.1. In the C_60_ case, *P*_γ_ is the zero-temperature distribution 
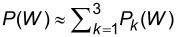
 broadened by a Lorentzian of width γ = 0.05 eV; *P*_γ_*_,_*_σ_ is obtained by convoluting *P*(*W*) with a Voigt distribution [18–19], whose Gaussian standard deviation σ = 0.172 eV and Lorentzian width γ = 0.068 eV are adjusted to some measurements on thick fullerene films. The experimental data are taken from [12–13] and plotted on the same arbitrary unit scale [18–19]. The theoretical distributions are computed with the DFT approach outlined in Section 2.1, using both the PBE and the B3LYP functionals. In the two examples of the Fermi gas, the zero-temperature work distributions are broadened with a Gaussian whose standard deviation δ_β_ corresponds to 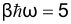, and compared with the analytical model of [32,35]. Vertical values are given in percent relative to the no-shake peak.

As shown in the perturbation model of [32,35], the role of the temperature is mostly accounted for by a Gaussian broadening characterized by the variance


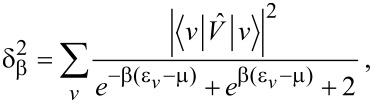


which is related to the particle–hole statistics, as well as to the diagonal matrix element of the external potential. In the fullerene case, another source of broadening is given by the core-hole lifetime. Besides, to cope with real photoemission experiments a further Gaussian term due to experimental uncertainties is needed [18–19]. Working at low thermal energies, we can therefore set 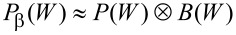, with *B*(*W*) denoting a broadening function, which includes the “thermal” Gaussian of standard deviation δ_β_. In Figure 6, we apply these considerations to determine the low-temperature profile of the work distributions for the core-ionized fullerene molecule and the shaken-up Fermi gas. Comparing the fullerene distribution with the experimental C 1s line shape from a thick C_60_ film, we find a significant match of the low-energy satellite structure, at excitation energies below about 4 eV, with the theoretical spectrum, apart from a peak position shift of 0.31 eV. As a further comparison, in Figure 6 (left panel) we report the work distribution obtained from the C_60_ and 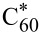 eigensystems with a three-parameter hybrid functional by Becke [51] (the B3LYP functional) instead of the PBE functional. Both the PBE and B3LYP results appear to be consistent within a peak position shift of about 1 eV, though the relative peak position structure of the experimental satellites seems to be better reproduced by the B3LYP functional. The Lorenztian broadening of the spectra are consistent with the calculated life-time broadening of the C 1s level in graphite [52].

On the other hand, the numerical shake-up response of the harmonically trapped Fermi gas is in excellent agreement with the analytical model presented in [32,35], in which a compact form was given to the characteristic function of work


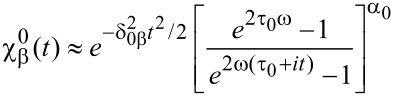


being the Fourier transform of the work distribution, and including all possible excited states, i.e., all possible components (Equaiton Equation 7). Two differences, inherent to the perturbation method, lie in the non-perfect periodic sequence of the shake-up peaks, which is significant only for low particle numbers, and in the critical exponent of the perturbation approach denoted α_0_ giving rise to the thermal broadening 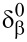. Accordingly, the effective temperatures corresponding to the numerical and analytical curves are different. When the perturbation series giving rise to 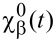 is summed over all orders, α_0_ will be eventually renormalized to α, and δ_0β_ to δ_β_.

## Conclusion

We have presented a numerical approach towards the calculation of the work distribution for a many-fermion system, shaken up by the sudden quench of a work parameter. To show the versatility of the method, we have discussed applications in two very diverse energy ranges, namely: (i) a fullerene molecule, where the absorption of a photon leads to a critical rearrangement of the ground state of the interacting valence electrons, witnessed by the Anderson orthogonality catastrophe; and (ii) a non-interacting gas of harmonically trapped fermions, where the catastrophe can be simulated in a controlled fashion by the appropriate embedding of a single probe qubit. We have suitably selected the parameters of the Fermi gas, in order to have roughly the same overall shake-up content as the fullerene molecule. In the plots of Figure 5 and Figure 6, we have explored the detailed features of the Anderson orthogonality catastrophe in the sequence of the shake-up satellites. The comparison with experiments on C_60_ indicates the reliability of the approach, putting emphasis on the present capability of DFT codes in predicting the excited state structure of molecules and solids [53]. On the other hand the comparison of the results from the trapped Fermi gas with the analytical model of [32,35] is suggestive of a deeper analysis into the definition of the critical exponent of the model, leaving open the possibility for further investigations in the weak-coupling regime.

## References

[1] Jarzynski C (2000). J Stat Phys.

[2] Jarzynski C (1997). Phys Rev Lett.

[3] Campisi M, Hänggi P, Talkner P (2011). Rev Mod Phys.

[4] Plastina F, Alecce A, Apollaro T J G, Falcone G, Francica G, Galve F, Lo Gullo N, Zambrini R (2014). Phys Rev Lett.

[5] Anderson P W (1967). Phys Rev Lett.

[6] Mahan G D (1967). Phys Rev.

[7] Nozières P, De Dominicis C T (1969). Phys Rev.

[8] Citrin P H, Wertheim G K, Baer Y (1977). Phys Rev B.

[9] Ohtaka K, Tanabe Y (1990). Rev Mod Phys.

[10] Karlsen T, Børve K J (2000). J Chem Phys.

[11] Sette F, Wertheim G K, Ma Y, Meigs G, Modesti S, Chen C T (1990). Phys Rev B.

[12] Enkvist C, Lunell S, Sjögren B, Svensson S, Brühwiler P A, Nilsson A, Maxwell A J, Mårtensson N (1993). Phys Rev B.

[13] Leiro J A, Heinonen M H, Laiho T, Batirev I G (2003). J Electron Spectrosc Relat Phenom.

[14] Gao B, Wu Z, Luo Y (2008). J Chem Phys.

[15] Petaccia L, Goldoni A, Lizzit S, Larciprete R (2005). J Electron Spectrosc Relat Phenom.

[16] Hentschel M, Guinea F (2007). Phys Rev B.

[17] Sindona A, Plastina F, Cupolillo A, Giallombardo C, Falcone G, Papagno L (2007). Surf Sci.

[18] Sindona A, Pisarra M, Naccarato F, Riccardi P, Plastina F, Cupolillo A, Ligato N, Caputi L S, Falcone G (2013). J Phys: Condens Matter.

[19] Sindona A, Naccarato F, Pisarra M, Riccardi P, Falcone G (2013). Thin Solid Films.

[20] Anderson P W, Yuval G (1969). Phys Rev Lett.

[21] Sindona A, Baragiola R A, Falcone G, Oliva A, Riccardi P (2005). Phys Rev A.

[22] Sindona A, Rudi S A, Maletta S, Baragiola R A, Falcone G, Riccardi P (2007). Surf Sci.

[23] Riccardi P, Pisarra M, Cupolillo A, Commisso M, Sindona A, Baragiola R A, Dukes C A (2010). J Phys: Condens Matter.

[24] Ubbelohde N, Roszak K, Hohls F, Maire N, Haug R J, Novotný T (2012). Sci Rep.

[25] Knap M, Shashi A, Nishida Y, Imambekov A, Abanin D A, Demler E (2012). Phys Rev X.

[26] Dóra B, Pollmann F, Fortágh J, Zaránd G (2013). Phys Rev Lett.

[27] Baeten M, Wouters M (2014). Phys Rev B.

[28] Campbell S, García-March M A, Fogarty T, Busch T (2014). Phys Rev A.

[29] Schiró M, Mitra A (2014). Phys Rev Lett.

[30] Ossipov A (2014). Phys Rev Lett.

[31] Goold J, Fogarty T, Lo Gullo N, Paternostro M, Busch T (2011). Phys Rev A.

[32] Sindona A, Goold J, Lo Gullo N, Lorenzo S, Plastina F (2013). Phys Rev Lett.

[33] Talkner P, Lutz E, Hänggi P (2007). Phys Rev E.

[34] Silva A (2008). Phys Rev Lett.

[35] Sindona A, Goold J, Lo Gullo N, Plastina F (2014). New J Phys.

[36] Heyl M, Kehrein S (2012). Phys Rev Lett.

[37] Schönhammer K, Gunnarsson O (1978). Phys Rev B.

[38] Brako R, Newns D M (1981). J Phys C: Solid State Phys.

[39] Doniach S, Sondheimer E H (1988). Green’s Functions For Solid State Physicists.

[40] Stevens W J, Basch H, Krauss K (1984). J Chem Phys.

[41] Krishnan R, Binkley J S, Seeger R, Pople J A (1980). J Chem Phys.

[42] Schmidt M W, Baldridge K K, Boatz J A, Elbert S T, Gordon M S, Jensen J H, Koseki S, Matsunaga N, Nguyen K A, Su S J (1993). J Comput Chem.

[43] Gordon M S, Schmidt M W, Dykstra C E, Frenking G, Kim K S (2005). Advances in electronic structure theory: GAMESS a decade later. Theory and Applications of Computational Chemistry, the First Forty Years.

[44] GAMESS.

[45] Perdew J P, Burke K, Ernzerhof M (1996). Phys Rev Lett.

[46] Perdew J P, Burke K, Ernzerhof M (1997). Phys Rev Lett.

[47] Pradhan N A, Liu N, Silien C, Ho W (2005). Phys Rev Lett.

[48] Bloch I, Dalibard J, Zwerger W (2008). Rev Mod Phys.

[49] Plastina F, Sindona A, Goold J, Lo Gullo N, Lorenzo S (2013). Open Syst Inf Dyn.

[50] Goold J, Busch T (2008). Phys Rev A.

[51] Becke A D (1993). J Chem Phys.

[52] Mele E J, Ritsko J J (1979). Phys Rev Lett.

[53] Pisarra M, Riccardi P, Sindona A, Cupolillo A, Ligato N, Giallombardo C, Caputi L (2014). Carbon.

